# New Insights into Improving the Photovoltaic Performance of Dye-Sensitized Solar Cells by Removing Platinum from the Counter Electrode Using a Graphene-MoS_2_ Composite or Hybrid

**DOI:** 10.3390/mi14122161

**Published:** 2023-11-26

**Authors:** Mozhgan Hosseinnezhad, Mehdi Ghahari, Ghazal Mobarhan, Mohsen Fathi, Arvydas Palevicius, Venkatramaiah Nutalapati, Giedrius Janusas, Sohrab Nasiri

**Affiliations:** 1 Department of Organic Colourants, Institute for Colour Science and Technology, Tehran P.O. Box 16765-654, Iran; 2 Department of Nanomaterials and Nanocoatings, Institute for Colour Science and Technology, Tehran P.O. Box 16765-654, Iran; maghahari@icrc.ac.ir (M.G.); gh.mobarhan@gmail.com (G.M.); 3 Department of Physics, Shahrood University of Technology, Shahrood P.O. Box 36155-316, Iran; kntu@aut.ac.ir; 4 Faculty of Mechanical Engineering and Design, Kaunas University of Technology, Studentu Street 56, LT 51373 Kaunas, Lithuania; arvydas.palevicius@ktu.lt (A.P.); giedrius.janusas@ktu.lt (G.J.); sohrab.nasiri@ktu.edu (S.N.); 5 Department of Chemistry, Faculty of Engineering and Technology, SRM Institute of Science and Technology, Kattankulathur 603203, Tamil Nadu, India; nvenkat83@gmail.com

**Keywords:** dye-sensitized solar cells, MoS_2_/graphene, efficiency, free-platinum, photovoltaic

## Abstract

Photovoltaic systems, such as dye-sensitized solar cells (DSSCs), are one of the useful tools for generating renewable and green energy. To develop this technology, obstacles such as cost and the use of expensive compounds must be overcome. Here, we employed a new MoS_2_/graphene hybrid or composite instead of platinum in the DSSCs. Furthermore, the correctness of the preparation of the MoS_2_/graphene hybrid or composite was evaluated by field emission scanning electron microscope (FESEM), and the results showed that the desired compound was synthesized correctly. Inexpensive organic dyes were used to prepare the DSSCs, and their chemical structure was investigated by density functional theory (DFT) and cyclic voltammetry (CV). Finally, the DSSCs were fabricated using MoS_2_/graphene composite or hybrid, and to compare the results, the DSSCs were also prepared using platinum. Under the same conditions, the DSSCs with MoS_2_/graphene composite illustrated better efficiency than MoS_2_/graphene hybrid or/and graphene.

## 1. Introduction

Population growth leads to an increase in energy demand in the areas of household consumption, industry, and technology. Therefore, it is necessary to develop new sources of energy [1,2]. Solar radiation is one of the energy sources that has the mentioned characteristics. Many studies have been conducted to harness this energy and effectively convert it into other energies, such as electrical energy [3,4]. One of the emerging energy generation technologies that uses sunlight is dye-sensitized solar cells (DSSCs) [5,6]. DSSCs are a very promising alternative to classical inorganic p–n junction solar cells as they combine molecular systems and nanoparticles to create a device that mimics photosynthesis, with the objective of turning sunlight into a renewable, reliable, and low-cost source of energy closer to existence [7]. The components of a DSSC are: photoanode, photosensitizers (dyes), electrolyte, and counter electrode. Two important challenges to the widespread development of this technology are the low efficiency and the high price of some of its components [8].

An important component that affects the cost of this technology is the counter electrode. Platinum is used to prepare the counter electrode, which is not only expensive but also an end-to-end resource [9]. Baskaran et al. considered the possibility of replacing platinum on the counter electrode with Cu_2_ZnSnS_4_/MoS_4_. The solar cell constructed with platinum had an efficiency of about 4.4%, while the efficiency with CZMO_8_ was 4.07%. The proximity of the results indicates the confirmation of the replacement of platinum with the prepared nanocomposite [10]. Sarkar et al. investigated the performance of DSSCs based on a new nanohybrid of CoNi_2_S_4_ graphene oxide as a counter electrode. The results showed that the DSSCs fabricated using RGO and CoNi_2_S_4_ have an efficiency of 3.44% and 5.78%, respectively. However, the use of the composite increases the efficiency up to 9.22%, which is in good agreement with the efficiency of platinum (9.51%) [11]. Research shows that different derivatives of graphene oxide can be a suitable option to replace platinum in the counter electrode.

**Figure 1:**
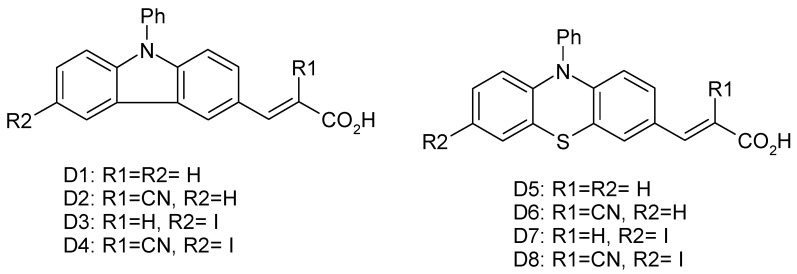
Chemical structure of photosensitizers.

In this paper, our aim was to investigate the replacement of platinum counter electrodes with new graphene-based compounds. For this purpose, a composite and a hybrid of graphene oxide (GO), MoS_2_, MoS_2_/GO nanohybrid, and MoS_2_/GO nano-composite were fabricated and used in a DSSC. To investigate the effect of the prepared compounds, solar cells containing both a Pt-free and a Pt counter electrode were fabricated. Organic dyes (Figure 1) and N719 were used as photosensitizers in the prepared cells. The process of synthesis of the organic dyes used as photosensitizers in this study has already been reported in ref. [12].

## 2. Experimental

### 2.1. Materials

The J-V curves were studied using the Bunko-Keiki CEP-2000 system and flower-like MoS_2_ was synthesized through the hydrothermal method following our previous work [13]. However, in the hybrid particles, the GO sheets were placed in a suspension of ammonium heptamolybdate and thiourea before the hydrothermal process. In this case, the MoS_2_ sheets formed on the graphene sheets.

### 2.2. X-ray Diffraction

The phase series was confirmed by X-ray diffraction (XRD) and a Philips XRD diffractometer using ${Cu}_{k\alpha }$ radiation at 40 KV, 30 mA, a step size of 0.05° (2ϴ), and a scan rate of 1°/min. X’Pert software was used for qualitative analysis and the report of the diffraction peak width (rad, β) at full width half maximum (FWHM) for different 2θ values according to the location of the peaks (version 4.9.0).

### 2.3. FESEM Analysis

FESEM is a magnification technique that allows magnification between ×10 and ×300,000, making the information about the elements present and their topographic data on the surface practically visible through an unlimited field depth. In this study, the MIRA3TESCAN-XMU FESEM was inserted into the high vacuum portion of the microscope through an exchange chamber and anchored to a moving stage. In the virtual FESEM, the object can be moved in the horizontal and vertical directions on the screen by pressing the arrows in the POSITION box. The detector types offered are the standard lens detector SE, the high-efficiency Everhart–Thornley detector (ETD) SE, and the angle-selective BSE detector. The in-lens detector SE is used to detect SE signals directly from the sample surface.

### 2.4. Electrochemical and Density Functional Theory (DFT) and Cyclic Voltammetry Study

The cyclic voltammetry (CV) technique was used to study the electrochemical properties of the synthesized dyes. The three electrodes were used to study the oxidation potential (E_ox_) in solution media. The counter and a Pt wire were chosen as counter and reference electrodes, respectively. The calibration routine in this technique was performed with the redox pair Fc and Fc^+^ [14,15]. Furthermore, the Gaussian software was utilized for density functional theory (DFT) calculations [16].

CV measurements of the organic dyes were carried out in acetonitrile. The oxidation potential (E_ox_) was measured using three small electrodes. A Ag quasi-reference electrode (QRE) was used as the reference. Platinum wires were used as the working and the counter electrodes. All electrode potentials were calibrated with respect to the ferrocene (Fc)/ferrocenium (Fc^+^) redox couplet. An acetonitrile solution (2 mL) of dyes containing tetrabutylammonium perchlorate (0.1 mol dm^−3^) and ferrocene (ca. 1 mmol dm^−3^) was prepared. The electrochemical measurements were performed at a scan rate of 100 mV s^−1^ [17].

### 2.5. Solar Cell Assembly

Nanocrystalline TiO_2_ paste was coated onto an FTO coated glass substrate. The dye was absorbed by dipping the coated glass for 18 h in an ethanolic solution of the organic dyes. Finally, the film was washed with an acetonitrile-ethanol 1:1 mixed solvent. Acenonitrile-ethylenecarbonate (*v/v* = 1:4) containing tetrabutyl ammonium iodide (0.5 mol dm^−3^) was used as electrolyte. Spray coating technique was employed to form GO, MoS_2_, GO/MoS_2_ nanohybrid, and GO/MoS_2_ nanocomposite thin films as the counter electrode. The dye-adsorbed photoelectrode, counter electrode, and the electrolyte solution were assembled into a sealed sandwich type solar cell. An action spectrum was measured under monochromatic light with a constant photon number (5 × 10^15^ photon cm^−2^ s^−1^) [18]. J-V characteristics were measured under illumination with AM 1.5 simulated sunlight (100 mW cm^−2^) through a shading mask (5.0 mm × 4 mm) by a Bunko-Keiki CEP-2000 system.

## 3. Results and Discussion

**Figure 2:**
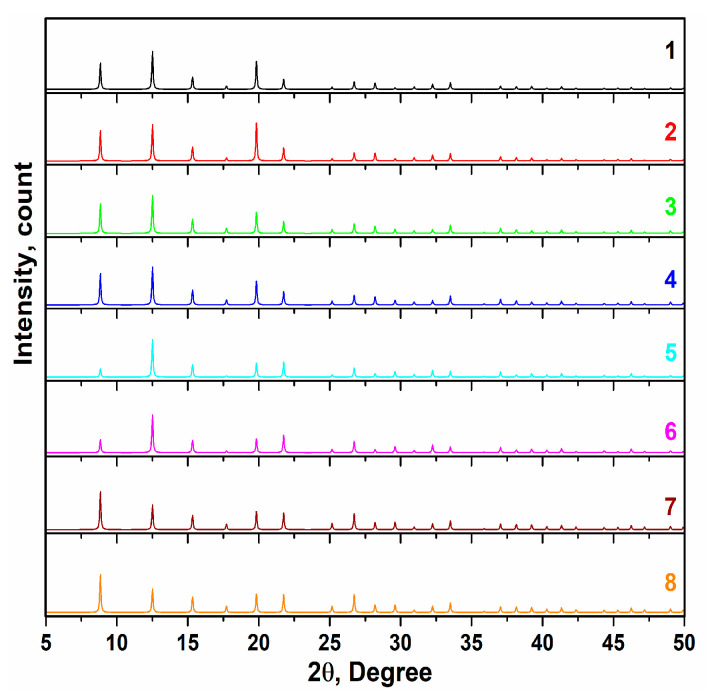
X-ray diffraction spectra of dyes 1–8.

**Figure 3:**
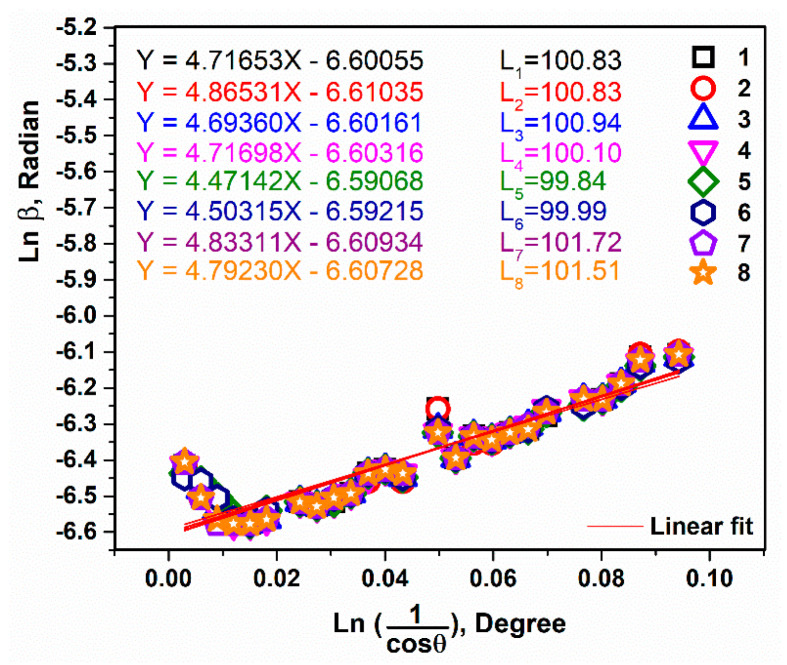
Linear fit plots of the Modified-Scherrer equation and the obtained intercepts to calculate crystallite size of dyes 1–8.

In the Modified-Scherrer method [19,20], when Ln β is plotted versus Ln ($\frac{1}{\cos\theta }$) and the least squares method is performed, the intercept gives Ln $\frac{K\lambda }{L}$, from which a single value for L can be obtained. $\frac{K\lambda {Cu}_{k\alpha 1}}{L}=e^{\left(\mathrm{intercept}\right)}$; in the equation, K is the shape factor (K = 0.89), $\lambda_{{Cu}_{k_{\alpha }}}$ = 0.15405 nm, L is the crystal size, and the intercept is related to the linear equation. According to the XRD spectra (Figure 2), no amorphous phases were observed and the sharp crystallite peaks belonged to 2θ ~12.4°, except for dyes 7 and 8, which were due to substitutions of the R1, R2, and phenothiazine derivative. Furthermore, the crystallite size values ranged from 99 to 101 nm (Figure 3).

**Figure 4:**
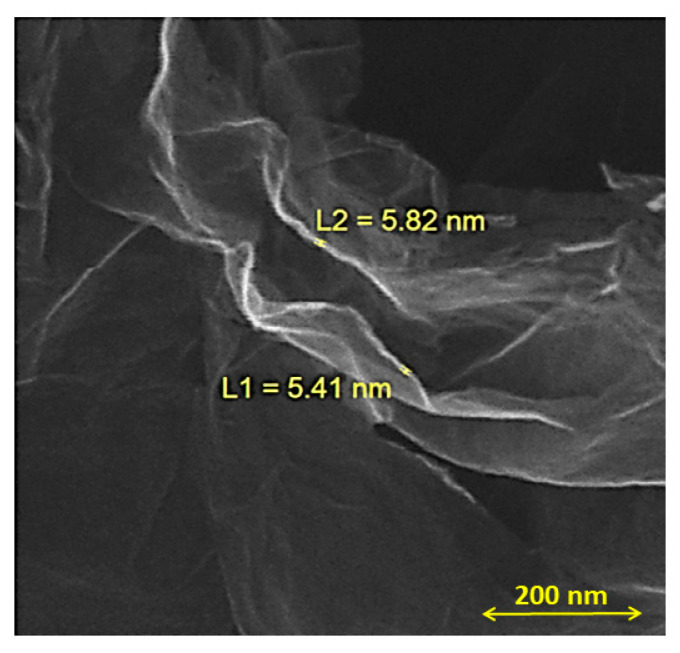
FESEM of the graphene flake.

**Figure 5:**
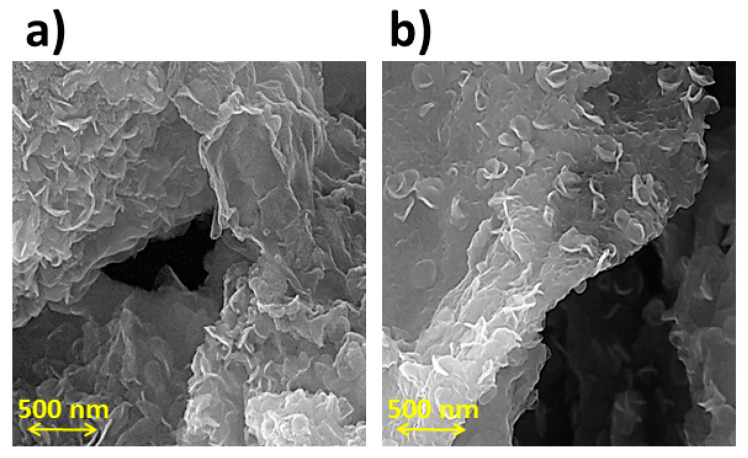
FESEM image of the MoS_2_/GO composite (**a**) and hybrid (**b**).

**Figure 6:**
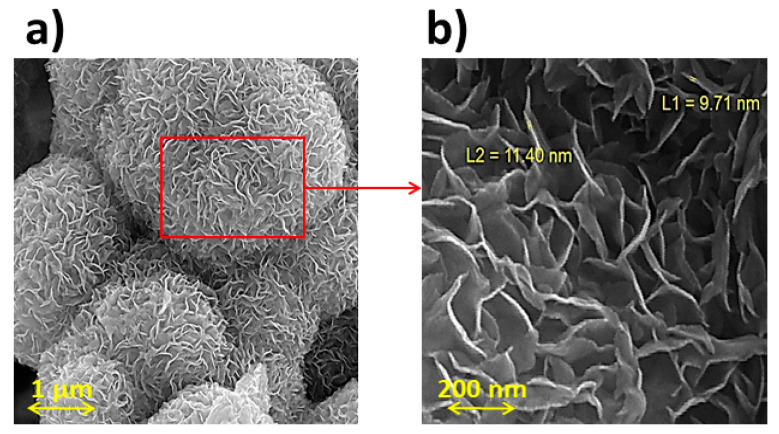
(**a**) FESEM micrographs of flower-shaped MoS_2_ nanoflakes on different scales and (**b**) zoom area.

The morphology of graphene was studied using FESEM. All graphene shows wrinkled and flexible nanosheets with a thickness below 20 nm. As shown in Figure 4, graphene exhibits a multilayered structure. Graphene flakes have a relatively large surface area and interconnected three-dimensional graphene sheets [21]. Figure 5 shows an overview of MoS_2_/GO composites and the hybrid morphology prepared by simple mixing and the hydrothermal method using surfactants, respectively. As shown in Figure 5, no isolated MoS_2_ particles or flakes can be seen in the MoS_2_/GO composites. In contrast, in the hybrid sample, the MoS_2_ layers are well distributed on the graphene surface [22]. Figure 6 shows microflowers of aggregated MoS_2_ nanosheets in monolayer form. It shows spherical particles composed of many layers of MoS_2_ nanosheets below 20 nm [23].

In contrast to thick graphitic material, atomically thin 2D materials such as grapheme have attracted much research interest as a possible platform for atomic diffusion barriers and ionic tunneling layers [24]. Recently, there have been rapid theoretical and experimental developments of elemental 2D materials in electrocatalytic reactions, including the hydrogen evolution reaction (HER), oxygen evolution reaction (OER), oxygen reduction reaction (ORR), carbon dioxide reduction (CO_2_ RR), and N_2_ reduction reaction (NRR). The most recent development in nanomaterials, especially 2D materials, provides enormous opportunities for high-performance solar cells beyond conventional bulk materials. Notably, some elemental 2D materials with high carrier mobility and tunable band gaps are considered to be promising candidates for solar cells. Many theoretical studies have investigated the potential of elemental 2D materials in solar cells [25]. Nikam et al. synthesized MoS_2_ nanosheets on three-dimensional (3D) conductive MoO_2_ via a two-step chemical vapor deposition (CVD) reaction. The 3D MoO_2_ structure can create structural disorders in MoS_2_ nanosheets (3D MoS_2_/MoO_2_). The MoS_2_ nanosheets could protect the inner MoO_2_ core from the acidic electrolyte in the HER. The high activity of the as-synthesized 3D MoS_2_/MoO_2_ hybrid material in HER is attributed to the small onset overpotential of 142 mV and a large cathodic current density of 85 mA cm^–2^ [26]. In this article, a series of novel compounds based on graphene have been used to prepare the counter electrode in the DSSCs.

**Figure 7:**
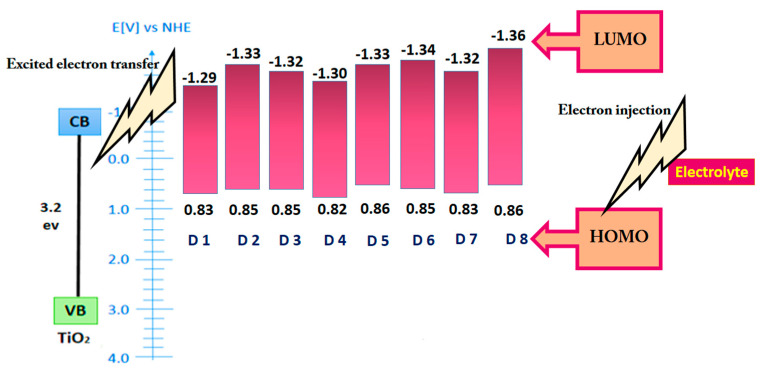
CV results of photosensitizers.

The CV method is used to measure the energy level of organic dyes on a laboratory scale. The information obtained by this method can be used to predict the behavior of dyes in the solar device [27,28]. The oxidation potential of peal in acetonitrile for dyes 1–8 was estimated to be +0.83, +0.85, +0.85, +0.82, +0.86, +0.85, +0.83, and +0.86 V vs. a normal hydrogen electrode (NHE), respectively. The LUMO level of the photosensitizers was obtained at −1.29, −1.33, −1.32, −1.30, −1.33, −1.34, −1.32, and −1.36 V versus NHE, respectively. The results confirm our ability to use dyes in DSSCs as photosensitizers (Figure 7). Silva et al. investigated the CV behavior of photosensitizers based on the tetrazole group. The results showed that the presence of strong donor electron groups concentrates the electron density of HOMO in addition to the effects of good adsorption on the semiconductor when di methyl formamide (DMF) solvent was used for this test [29]. The compounds used in this study dissolved well in acetonitrile, so this solvent was used for the CV test. However, the results showed that the compounds used were capable of transferring electrons to the semiconductor layer and accepting electrons from the electrolyte. Subsequently, the laboratory results were compared with the theoretical studies.

The catalytic activity of the electrode materials was examined by cyclic voltammetry (CV) measurements. The results showed that two pairs of redox peaks can be observed, with the negative pair belonging to redox reaction (1) and the positive pair belonging to redox reaction (2):

(1) $$I_{3}^{-}+2e^{-}\to 3I^{-}$$

(2) $$3I_{2}+2e^{-}\to 2I_{3}^{-}$$

The shape of the measured CV curves is similar to those observed in Pt CE, indicating a similar electrochemical behavior to Pt CEs. For new materials, the current density of the CV curve increased significantly, indicating higher catalytic activity. The improvement of the electrocatalytic activity can be partially ascribed to the overall surface area that leads to more diffusion pathways for the electrolyte species [30,31].

**Figure 8:**
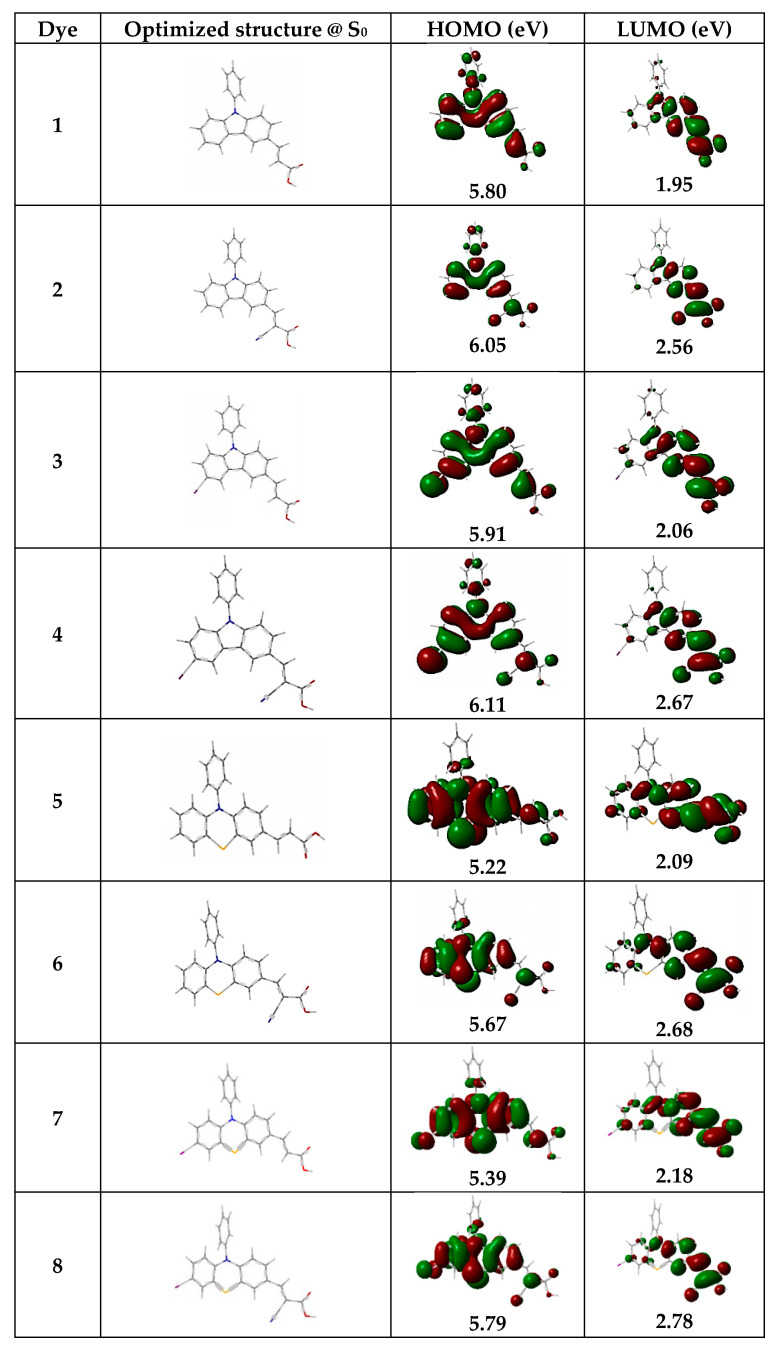
The optimized molecules @ S_0_ and HOMO–LUMO distribution of the dyes.

All calculations of the derivatives were performed using the Gaussian 09 program package [32]. Quantum chemical calculations were performed and complete optimization of all structures was done using a DFT method coupled to the TD-DFT/Becke-three-Lee-Yang-Parr (B3LYP) hybrid/6-31g(d,p) level of theory [33]. No symmetry constraints were used in the optimizations. To verify the nature of the stationary geometries, a harmonic vibration frequency calculation was carried out to ensure that no imaginary frequencies were located. Taking into account DFT calculations, the distributions of the highest occupied molecular orbital (HOMO) and lowest unoccupied molecular orbital (LUMO), as well as the orbitals of the transitions, were performed when the carbazole and/or phenothiazine derivatives were coupled to the phenyl ring. Figure 8 illustrates the HOMO, LUMO, and S_0_ of the organic photosensitizers. The amounts of HOMO ranged from −5.22 to −6.11 eV for the dyes in tandem. Dye 4 showed the highest value of the HOMO distribution (6.11 eV), and, in comparison, it is due to the chemical structure that the simultaneous substitution of 2-cyanoacrylic acid (C_4_H_3_NO_2_) and iodide (I) in the carbazole derivative increases the HOMO value. Moreover, HOMO shows that the introduction of groups at these sites has a remarkable effect on the orbital properties of the molecule, affecting both the energy band gap and the photovoltaic properties. In addition, LUMO values of −1.95, −2.56, −2.06, −2.67, −2.09, −2.68, −2.18, and −2.78 eV were calculated for dyes 1–8, respectively. The special substitution was performed in the LUMO region, and the simultaneous use of C_4_H_3_NO_2_ and I on the phenothiazine derivative showed that the LUMO shifted to a higher energy level of 2.78 eV. To study and predict the interactions with the solid surface, conceptual density function theory (CDFT), i.e., electronic chemical potential μ (Equation (3)), chemical hardness η (Equation (4)), and global electrophilicity ω (Equation (5)), has been widely used to study and explain the reactivity of organic dyes [33,34].


(3) $$\mu =\frac{E_{\mathrm{HOMO}}+E_{\mathrm{LUMO}}}{2}$$

(4) $$\eta =E_{\mathrm{LUMO}}-E_{\mathrm{LUMO}}$$

(5) $$\omega =\frac{\mu^{2}}{2\eta }$$

The calculated values of μ, η, and ω are listed in Table 1. According to the results in Table 1, the μ of dye 5 (−3.65 eV) is higher than that of the other dyes. Therefore, throughout the adsorption process, the electron transfer ratio is higher for dye 5 and has the ability to evade the orientation of the electrons. Consequently, dye 5 behaves as a nucleophile, while dye 4 reacts as an electrophile, as is confirmed by the higher HOMO value of dye 4. In terms of the ω index, dye 8 exhibits high electrophilicity values and therefore has a high reactivity of nucleophiles [35]. According to the η values, dye 8 is the softest among the dyes, while dyes 1 and 3 are the hardest. Therefore, dye 8 has a low excitation energy and the electron density is easy to change, while the large excitation energy or electron density of dyes 1 and 3 is very difficult to change [36].

**Figure 9:**
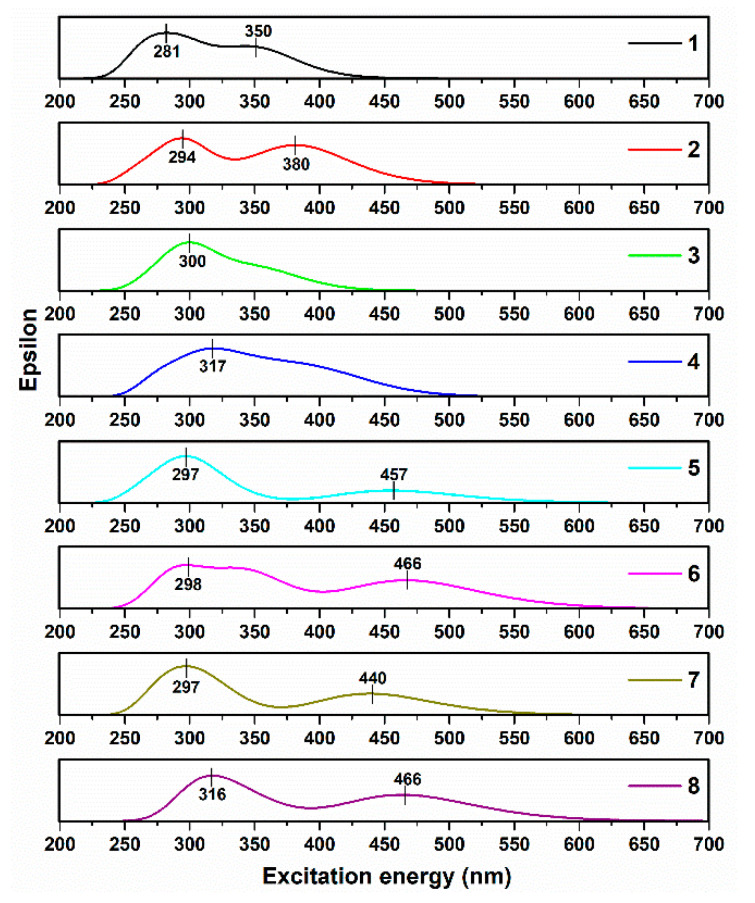
Simulated absorption spectra at TD-DFT/B3LYP/6-31g(d,p) of dyes.

Furthermore, the E_H-L_ values were recorded as 3.85, 3.49, 3.85, 3.44, 3.13, 2.99, 3.21, and 3.01 eV for dyes 1–8, respectively. It is obvious that C_4_H_3_NO_2_ and I as substituents simultaneously on the phenothiazine derivative decrease the E_H-L_ value. TD-DFT results were carried out with optimized S_0_ geometries and considering the solvation effects of dichloromethane using the C-PCM model [33,37]. TD-DFT uses different functions for the interpretation of absorption molecule spectra [38,39]. Moreover, DFT functions were used to measure the absorption behavior and compare it with experimental data. Taking the transitions into account, the theoretical UV-Vis spectrum was determined at the relativistic level using Gaussian software (Figure 9). The maximum wavelength values extracted from the calculations of DFT agreed well with the experimental maximum wavelengths of the dyes. It is clear that dyes 6 and 8 showed visible spectra in the range from 400 to 600 nm, because the LUMO values of these dyes were higher due to the lowest optical bandgap energy (evaluated from the edges of the theoretical UV-Vis of the dyes, $E_{g_{\mathrm{opt}}}=\frac{1240}{\lambda_{\mathrm{onset}}}$). In an overall comparison of these groups, it was noticed that the HOMO values were higher when the carbazole derivative was chosen as the HOMO part rather than the phenothiazine derivative. It was interesting to note that using the phenothiazine derivative as the HOMO part of the absorption spectrum showed a better visible range as dyes 5, 6, 7, and 8 showed maximum wavelength values of 457, 466, 440, and 466 nm in the visible range.

We overlay with the CIE photopic spectral luminous efficiency function, which represents the average spectral sensitivity of human visual perception to light. The average visible light transmission (AVT) through the photoanode was calculated based on the following equation (Equation (6)) [40]:

(6) $$\mathrm{AVT}(\%)=\frac{\int_{390}^{880}T(\lambda )\times V(\lambda )\times S(\lambda )d\lambda }{\int_{390}^{880}V(\lambda )\times S(\lambda )d\lambda }$$

where T(λ) is the transmission of the sample, V(λ) is the photopic spectral luminous efficiency function, and S(λ) is the AM 1.5 g solar spectrum [34]. AVTs (%) of dyes 1–8 are 83.7, 82.5, 82.6, 88.1, 81.6, 85.6, 88.6, and 87.9%, respectively. Thus, all dyes are very promising candidates as photosensitizers for highly transparent DSSCs. This trend in AVT values could be in contrast with the fact all dyes have a good molar attenuation coefficient (ε) in solution [40,41].

In this study, titanium dioxide was used as the semiconductor in the photoanode. Titanium dioxide is one of the best metal oxides for use in DSSCs, and its optimization and restructuring have been the subject of numerous studies [42]. However, one of the most important and influential components of the cost of a DSSC is the counter electrode. In this research, new compounds were used as the counter electrode and their performance was investigated in comparison with platinum. The organic dyes used as photosensitizers in DSSCs are based on indoline (dyes 1–8). Platinum was used as a counter electrode in the fabrication of solar cells with these compounds. The efficiencies obtained for dyes 1 to 8 and N719 in the structure of the platinum DSSCs are: 2.98, 3.69, 4.31, 5.87, 2.11, 2.95, 3.71, 3.98, and 9.48%. To remove the expensive platinum, graphene oxide (GO), MoS_2_, GO-MoS_2_ nanohybrid, and GO-MoS_2_ nanocomposite were investigated. The photovoltaic performance of each DSSC device with TiO_2_ is summarized in Table 2, Table 3, Table 4 and Table 5. Furthermore, the results show that the performance of the photosensitizers containing cyanoarylic acid is better than that of the compounds lacking this substitution. The level of photocurrent depends on the chemical structure of the dye and the ability to bind effectively with the semiconductor [43,44]. Consequently, a decrease in photocurrent indicates poor bonding between the dye and the nanosheet [45].

Graphene oxide has poor electrocatalytic properties that reduce the efficiency and FF values of DSSCs. For example, the efficiency of a dye 6 in a DSSC with a platinum counter electrode is about 5.87%, but this value is reduced to 64% by changing the counter electrode to graphene oxide, which is about 3.93%. Suriani et al. used graphene oxide and its hybrid with platinum to fabricate a solar cell. They used the spray coating technique to fabricate a thin film. Moreover, the efficiency of the DSSCs with graphene oxide as the counter electrode is about 23% of that of DSSCs with platinum as the counter electrode. It is important to note that the use of a hybrid of graphene oxide and platinum improves the efficiency of the DSSCs by 5% compared to the platinum-individual DSSCs [46,47]. In another study, Tamilselvi et al. investigated the effects of using graphene oxide and its hybrid with NiSe_2_. The results showed that the performance of the DSSCs was improved by about 57% in the presence of the graphene oxide-NiSe_2_ hybrid [48]. Due to the significant reduction of efficiency in the presence of graphene oxide as a counter electrode of DSSCs found in this and other studies, the use of a hybrid or composite of graphene oxide seems necessary. One of the proposed components to optimize the performance of graphene oxide in DSSCs is MoS_2_. Francis et al. fabricated a new configuration of MoS_2_ using a hydrothermal process. The morphological structure of the desired composition was confirmed by XRD and FESEM methods. Further, a spin coating method was used to fabricate the counter electrode of MoS_2_. The efficiency of the DSSCs fabricated with this counter electrode was about 4.50% [49]. Silambarasan et al. prepared a composite using a combination of graphene oxide and MoS_2_ as a counter electrode for DSSCs. The efficiencies of DSSCs containing platinum, MoS_2_, and the fabricated composite were 5.17, 2.01, and 4.65%, respectively. As can be seen, the efficiency of composite-based DSSCs is very close to that of platinum [50]. The use of MoS_2_ to fabricate a suitable hybrid or composite of graphene oxide is a new approach to reducing the cost of DSSC technology. In this study, a hybrid or composite of graphene oxide and MoS_2_ was prepared as a counter electrode for use in DSSCs. The results showed that the efficiency of DSSCs using hybrids and composites is similar to platinum up to 95%. In other words, these compounds can replace platinum.

Although PCE and AVT are often inversely related, as discussed, all photovoltaic technologies have potential applications. Progressive development warrants new compound figures of merit beyond PCE and AVT that allow for improvements to be referenced across various technologies. Light utilization efficiency (LUE) metric was calculated by Equation (7) [41].

(7) LUE=PCE×AVT

This metric enables comparison between technologies against theoretical limits and also represents an overall system efficiency: for example, in a window or display application, this would be the combination of generated power efficiency and overall lighting efficiency (that is, the transmitted light per incident lighting power). A device with a high LUE could be applied over a window to provide power without blocking natural light from entering the room to reduce the need for artificial lighting during daylight hours [40]. The LUE value obtained for DSSCs was in the range of 41–44%.

## 4. Conclusions

In this research, two types of hybrids and composites were prepared from the combination of graphene oxide and MoS_2_ for use in the counter electrode of DSSCs by the hydrothermal method. All graphene shows wrinkled and flexible nanosheets with thickness below 20 nm. No isolated MoS_2_ particles or flakes can be seen in the MoS_2_/GO composites. In contrast, in the hybrid sample, the MoS_2_ layers are well distributed on the graphene surface. The LUMO level of the photosensitizers was obtained at −1.29, −1.33, −1.32, −1.30, −1.33, −1.34, −1.32, and −1.36 V versus NHE, respectively. However, the results showed that the compounds used were capable of transferring electrons to the semiconductor layer and accepting electrons from the electrolyte. The results show that the conductivity characteristics of hybrid and composite GO/MoS_2_ prepared from their components (GO and MoS_2_) are higher and can be a suitable option to replace platinum in DSSCs. Graphene oxide has poor electrocatalytic properties that reduce the efficiency and FF values of DSSCs. Eight dyes based on indoline and N719 as a standard dye were used as sensitizers in the DSSCs containing crystallite size values ranging from 99 to 101 nm. The AVTs (%) of dyes 1–8 are 83.7, 82.5, 82.6, 88.1, 81.6, 85.6, 88.6, and 87.9%, respectively. Thus, all dyes are very promising candidates as photosensitizers for highly transparent DSSCs. Finally, the DSSCs were prepared and evaluated using organic dye, a titanium dioxide photoelectrode, and five different counter electrodes (GO, MoS_2_, GO-MoS_2_ nano-hybrid, and GO-MoS_2_ nano-composite, Pt). The results showed that the efficiency of DSSCs using hybrids and composites is similar to platinum up to 95%. In other words, these compounds can replace platinum. The LUE value obtained for DSSCs was in the range of 41–44%.

## Figures and Tables

**Table 1:** Calculated quantum chemical parameters of dyes.

**Dye**	**1**	**2**	**3**	**4**	**5**	**6**	**7**	**8**
**μ (eV)**	−3.87	−4.30	−3.98	−4.39	−3.65	−4.17	−3.78	−4.28
**η (eV)**	3.85	3.49	3.85	3.44	3.13	2.99	3.21	3.01
**ω (eV)**	1.95	2.65	2.06	2.80	2.13	2.91	2.23	3.05

**Table 2:** Photovoltaic data of DSSCs containing GO as counter electrode.

Dye	J_SC_ (mA·cm^−2^)	V_OC_ (V)	FF	η (%)
**1**	5.12	0.45	0.46	1.06
**2**	6.16	0.51	0.42	1.32
**3**	7.59	0.53	0.44	1.77
**4**	9.09	0.52	0.44	2.08
**5**	3.08	0.54	0.44	0.73
**6**	4.81	0.51	0.44	1.08
**7**	3.86	0.51	0.66	1.30
**8**	5.92	0.54	0.45	1.44
**N719**	12.92	0.68	0.42	3.69

**Table 3:** Photovoltaic data of DSSCs containing MoS_2_ as counter electrode.

Dye	J_SC_ (mA·cm^−2^)	V_OC_ (V)	FF (%)	η (%)
**1**	7.29	0.57	0.63	2.62
**2**	9.04	0.64	0.60	3.47
**3**	10.28	0.62	0.64	4.08
**4**	12.62	0.64	0.64	5.17
**5**	4.39	0.65	0.64	1.83
**6**	9.01	0.55	0.45	2.23
**7**	7.73	0.65	0.65	3.27
**8**	8.16	0.66	0.65	3.50
**N719**	16.68	0.84	0.62	8.68

**Table 4:** Photovoltaic data of DSSCs containing GO-MoS_2_ nano-composite as counter electrode.

Dye	J_SC_ (mA·cm^−2^)	V_OC_ (V)	FF (%)	η (%)
**1**	8.17	0.55	0.63	2.83
**2**	9.52	0.62	0.61	3.60
**3**	10.30	0.63	0.65	4.22
**4**	14.39	0.62	0.64	5.71
**5**	4.90	0.63	0.65	2.01
**6**	7.13	0.62	0.64	2.83
**7**	8.73	0.62	0.65	3.52
**8**	8.87	0.64	0.66	3.75
**N719**	18.65	0.81	0.62	9.37

**Table 5:** Photovoltaic data of DSSCs containing GO-MoS_2_ nano-hybrid as counter electrode.

Dye	J_SC_ (mA·cm^−2^)	V_OC_ (V)	FF (%)	η (%)
**1**	7.71	0.57	0.63	2.77
**2**	9.40	0.62	0.61	3.558
**3**	9.98	0.65	0.64	4.15
**4**	13.67	0.64	0.64	5.60
**5**	4.71	0.65	0.64	1.96
**6**	6.69	0.64	0.64	0.74
**7**	8.32	0.64	0.65	3.46
**8**	8.64	0.66	0.65	3.71
**N719**	17.40	0.85	0.62	9.17

## Data Availability

Data are contained within the article.
